# Comparison of the ocular surface microbiota between thyroid-associated ophthalmopathy patients and healthy subjects

**DOI:** 10.3389/fcimb.2022.914749

**Published:** 2022-07-26

**Authors:** Xuan Ji, Kui Dong, Ji Pu, Jing Yang, Zhaoxia Zhang, Xiaoling Ning, Qin Ma, Zhiming Kang, Jianguo Xu, Bin Sun

**Affiliations:** ^1^ Shanxi Eye Hospital, Shanxi Province Key Laboratory of Ophthalmology, Taiyuan, China; ^2^ State Key Laboratory of Infectious Disease Prevention and Control, National Institute for Communicable Disease Control and Prevention, Chinese Center for Disease Control and Prevention, Research Units of Discovery of Unknown Bacteria and Function (2018 RU010), Chinese Academy of Medical Sciences, Beijing, China

**Keywords:** high-throughput sequencing, ocular surface, ocular surface microbiota, thyroid-associated ophthalmopathy, bacteria

## Abstract

**Purpose:**

Thyroid-associated ophthalmopathy (TAO) is a chronic autoimmune disease. In this study, high-throughput sequencing was used to investigate the diversity and composition of the ocular microbiota in patients with TAO.

**Methods:**

Patients with TAO did not receive treatment for the disease and did not have exposed keratitis. Patients with TAO (TAO group) and healthy individuals (control group) were compared. All samples were swabbed at the conjunctival vault of the lower eyelid. The V3 to V4 region of the 16S rDNA was amplified using polymerase chain reaction and sequenced on the Illumina HiSeq 2500 Sequencing Platform. Statistical analysis was performed to analyze the differences between the groups and the correlation between ocular surface microbiota and the disease. The ocular surface microbiota of patients and healthy individuals were cultured.

**Results:**

The ocular surface microbiota structure of TAO patients changed significantly. The average relative abundance of *Bacillus* and *Brevundimonas* increased significantly in the TAO group. *Corynebacterium* had a significantly decreased relative abundance (P<0.05). *Paracoccus*, *Haemophilus*, *Lactobacillus*, and *Bifidobacterium* were positively correlated with the severity of clinical manifestations or disease activity (P<0.05). *Bacillus cereus* and other opportunistic pathogens were obtained by culture from TAO patients.

**Conclusions:**

This study found that the composition of ocular microbiota in patients with TAO was significantly different from that in healthy individuals. The ocular surface opportunistic pathogens, such as *Bacillus*, *Brevundimonas*, *Paracoccus*, and *Haemophilus* in TAO patients, increase the potential risk of ocular surface infection. The findings of this study provide a new avenue of research into the mechanism of ocular surface in TAO patients.

## Introduction

The human microbiome is the sum of genetic, metabolic, and structural substances of microorganisms in the human body (Human Microbiome Project, 2012). In the human body, microbiome studies have found that the oral cavity, skin, nasal cavity, digestive tract, and birth canal have their own unique microbial communities and can play a vital role in human health and disease (Cho and Blaser, 2012). Studies have found that bacteria exist on the eye surfaces of both healthy individuals and those with diseases. The application of high-throughput sequencing to the study of microbiota provides more opportunities for understanding the structure and function of the ocular surface microbiota. Studies have found that some bacteria in healthy ocular surface microbiota can participate in immune regulation and exert a protective effect by inhibiting the propagation of pathogenic bacteria. For example, healthy ocular surface microbiota can enhance immunoglobulin A secretion and neutralize ocular surface antigens (Kugadas and Gadjeva, 2016; Kugadas et al., 2017). *Corynebacterium mastitidis* can induce interleukin-17 production and inhibit the growth of *Candida albicans* or *Pseudomonas aeruginosa* by stimulating T cells in the mucosa of healthy human eyes (St Leger et al., 2017). In the meantime, studies have shown that ocular bacteria may be involved in the occurrence and development of various eye diseases. Patients with dry eyes (Li, Gong, et al., 2019), severe meibomian gland dysfunction (Dong et al., 2019), traumatic corneal ulcer (Kang et al., 2020), and diabetes (Li, Yi, et al., 2019) all experience changes in ocular surface microbiota, which may result in decreased abundance of protective microbiota, enabling pathogenic bacteria and opportunistic pathogens to occupy a dominant position.

Thyroid-associated ophthalmopathy (TAO) is one of the most common orbital disorders in adults. It is generally accepted that TAO is a chronic autoimmune disease. The thyrotropin receptor expressed by orbital fibroblasts and fat cells are the target of intraorbital autoimmunity, and the anti–thyrotropin-receptor antibodies are generated during the cellular immunity process (Bahn, 2010). These antibodies, together with secreted type 1 helper T cytokines on-γ and tumor necrosis factor, initiate the organizational changes of TAO, which can lead to pathological changes, such as lymphocyte infiltration, periorbital tissue edema, myofibroblast differentiation, and adipocyte proliferation (Bahn, 2010; Smith and Hegedüs, 2016). Under the stimulation of immuno-inflammatory response, the early manifestations of active TAO include photophobia, tearing, foreign body sensation, eyelid swelling, conjunctival hyperemia, and edema (Bartalena et al., 2021). With the development of the disease, eyelid retraction, exophthalmia, and eye movement disorder may occur, increasing the area of the ocular surface exposed to air (Bartalena et al., 2021). The exacerbation of these symptoms can lead to exposure keratitis, causing severe corneal damage and even threatening vision (Bartalena et al., 2021). As can be seen, the pathological mechanism and clinical manifestations of TAO suggest that the disease may alter the ocular surface microbiota of patients. These changes have been confirmed in the study of exposure keratitis in patients with TAO, and various pathogenic bacteria and opportunistic pathogens have been cultured (Naik et al., 2019). However, the changes in ocular surface microbiota of TAO patients without exposed keratitis remain to be further explored.

Therefore, in this study, high-throughput sequencing technology was used to explore the diversity and composition of ocular surface flora in TAO patients without exposed keratitis.

## Materials and methods

### Study design and population

A total of 70 TAO patients without exposure keratitis and 77 healthy volunteers were recruited from 2019 to 2022 during their first visit to Shanxi Eye Hospital. At the same time, 13 unsampled swabs were retained during the sampling process as blank controls for the environment.

Thyroid function of TAO subjects was normal or elevated, and some subjects received oral methimazole or propylthiouracil to control their thyroid function. All participants had not previously received eye drops, glucocorticoid therapy, oral immunosuppressants, or radiotherapy. All patients were examined by the same ophthalmologist to evaluate clinical symptoms, orbital computed tomography, and Tc^99m^-DTPA scintigraphy for TAO diagnosis (Sun et al., 2017). Clinical symptoms included eyelid withdrawal, exophthalmia, and corneal epithelial injury based on the European Group guidelines on Graves’ orbitopathy (Bartalena et al., 2008) and supplemented by the diagnosis of dry eyes (Clayton, 2018). Patient activity assessment was based on the TAO Clinical Activity Score (CAS), and severity assessment was based on the European Group guidelines on Graves’ orbitopathy (Bartalena et al., 2008).

Exclusion criteria were severe systemic diseases; pregnant or breastfeeding women; antibiotic, anti-inflammatory, and corticosteroid eye drop administration within 3 months; wearing of contact lenses; sinusitis within 3 months; history of eye surgery or trauma; and other eye diseases. Volunteers in the control group were required to complete medical history collection and eye specialty examination to exclude TAO and the exclusion criteria.

This study was approved by the hospital ethics committee. Written informed consent was obtained from all patients in accordance with the Helsinki Declaration (SXYYLL20190320).

### Sample collection and culture

Samples were collected by qualified ophthalmologists in a clean disposal room following the established procedures in batches. Before sampling, hands were disinfected, and disposable gloves were worn. Subsequently, the patients were subjected to local eye anesthesia with proparacaine hydrochloride eye drops (Alcon-Couvreur, Bornem, Belgium). After 1–3 min, the patients were instructed to look upward, and a sterile swab (4N6FLOQSwabs; COPAN Diagnostics, Murrieta, CA) was wiped from the inner canthus of the patient’s subconjunctival fornix to the outer canthus twice. At the end of each sampling batch, sterile swabs from the same production batch without nucleic acid sampling were used as blank control samples.

The sample swab was sealed in a sterile centrifuge tube. Swabs from all subjects were placed in ice boxes and sent to the laboratory for storage at -80°C for centralized nucleic acid extraction. Genomic DNA was extracted from all samples using the MO-Bio PowerSoil^®^ Kit (QIAGEN, Hilden, Germany).

At the same time, swabs from the other eye of 43 TAO patients and healthy individuals were placed in a bacteria-preserving solution for conventional culture. After enrichment, those samples were inoculated in Brain-Heart Infusion medium (OXOID, UK) supplemented with sheep blood and cultured in oxygen (30°C), 5% carbon dioxide (35°C), and anaerobic (35°C) environment. The approximate full-length 16S rRNA gene sequence was returned by sequencing (RuiBiotech, Beijing, China) and was compared online with the GeneBank database (https://www.ncbi.nlm.nih.gov).

### Polymerase chain reaction and qualification

Specific primers (341F-806R) were used to amplify 16S rRNA in the corresponding regions (16S V3-V4). Polymerase chain reaction (PCR) was performed using approximately 10 ng of template DNA. The PCR process included 15 μL of Phusion High-Fidelity PCR Master Mix (New England Biolabs, Ipswich, MA) and 0.2 μM of forward and reverse primers. Thermal cycling consisted of initial denaturation at 98°C for 1 min; 30 cycles of denaturation at 98°C for 10 s, annealing at 50°C for 30 s, and elongation at 72°C for 30 s; and final extension at 72°C for 5 min. The PCR products were mixed in equimolar ratios. Purified PCR products were extracted using the Qiagen Gel Extraction Kit (Qiagen). The TruSeq^®^ DNA PCR-Free Sample Preparation Kit (Illumina, San Diego, CA) was used to generate the sequencing library and add indexes. Library quality assessment was performed using the Qubit@2.0 Fluorometer (Thermo Fisher Scientific, Waltham, MA) and Agilent Bioanalyzer 2100 (Agilent, Santa Clara, CA). Library sequencing was performed on the NovaSeq platform (Illumina).

### Data analyses of clinical samples and statistical analyses

Paired-end reads were assigned to samples based on their unique barcode. Unique barcode and primer sequences were truncated. FLASH (v1.2.7, http://ccb.jhu.edu/software/FLASH/) was used to read and merge paired-end reads. QIIME (v1.9.1, http://qiime.org/scripts/split libraries fastq.html) was used for quality filtering of raw tags. The UCHIME algorithm (UCHIME Algorithm, http://www.drive5.com/usearch/manual/uchime algo.html) was used to detect and remove chimeric sequences after comparing tags with the reference databases (SILVA database. Tags were clustered to obtain the operational taxonomy unit (OTU) sequence. Then, the tags were compared with the OTU sequence to obtain the OTU abundance distribution table for each sample. Mothur software was used for species annotation analysis using the SILVA database, and the confidence threshold was set at 0.8. Due to the low biomass of eye surface samples, environmental contamination can be easily introduced in sample collection, DNA extraction, library construction, and sequencing. Blank control samples were used as the standard to monitor the contamination of nucleic acid extraction samples in the same batch. By treating specific microorganisms in blank control samples as spike-ins, we estimated sample contamination and excluded three samples from the TAO group and 13 samples from the control group from subsequent analysis. The accession code of the microbiome raw sequence data in the NCBI Sequence Read Archive is PRJNA807746 (SRR18065410-SRR18065514).

Statistical analysis was performed using QIIME2 and R. Alpha diversity was evaluated using Shannon’s diversity index. Beta diversity was revealed by Bray-Curtis dissimilarity. Differences between groups were analyzed using Wilcoxon rank-sum test and linear discriminant analysis (cutoff = 4.0). Intra-group differences were analyzed using t-test dichotomous traits and t-test regression coefficients. Spearman’s correlation coefficients were used to assess the correlation between microbiota and disease. *P*< 0.05 represents statistical significance.

## Results

### Bacterial diversity

**Figure 1:**
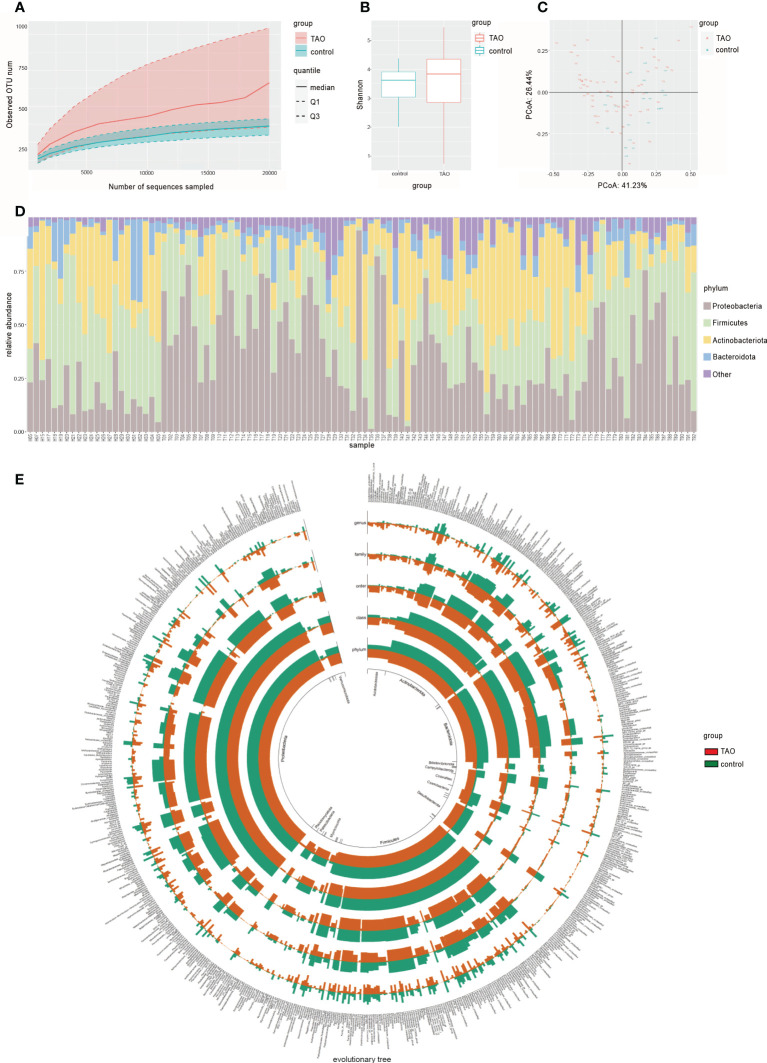
**(A)** Rarefaction curves of TAO and control samples. The median, and quartile values of the two groups of samples are shown. **(B)** Alpha diversity (Shannon index) and **(C)** beta diversity (principal coordinate analysis) are used to measure the diversity and differences between the two groups. **(D)** Dominant ocular surface microbiota at the phylum level and **(E)** evolutionary tree are indicated by different colors.

A total of 89 ocular surface microbe samples were included (TAO, n=67; control, n=22), yielding 9,059,610 sequences (**Table 1**). After removing all low-quality and non-bacterial 16S rRNA reads, 6,709,351 reads remained. An average of 60,994 reads for each sample was retained for further analysis. A total of 4899 OTUs were obtained in the two groups, including 1745 and 4711 OTUs in the control and TAO groups, respectively. The number of OTUs in the TAO group was higher than that in the control group, suggesting that the bacterial load was higher. The dilution curve directly reflects the rationality of the sequencing data and indirectly reflects the richness of species in the samples (**Figure 1A**). To reflect the richness and diversity of microbial communities in the samples, alpha diversity analysis was performed on all samples under a 97% consistency threshold by comparing the structural similarity between the TAO and control groups (**Figure 1B**). The results suggested that there was no significant difference in alpha diversity (P=0.34). The diversity of all samples was demonstrated based on principal coordinate analysis, showing no significant aggregation difference between the two groups (**Figure 1C**).

**Table 1:** Features of Subjects.

	TAO	Control
**sequencing**		
No. of sample(subject)	67(70)	22(77)
Average age	44.25 ± 13.44	62.64 ± 7.11*
Male/female	8/39	7/15
**culture**		
No. of sample(subject)	43(70)	43(77)
Average age	42.86 ± 14.97	32.49 ± 4.80*
Male/female	16/27	25/18

TAO, thyroid-associated ophthalmopathy; Control, healthy volunteers; data are the mean ± SD; *P < 0.05.

### Taxonomic assignment

A total of 34 phyla and 756 genera of bacteria were identified in the TAO and control groups. At the phylum level, the composition of dominant bacteria on the ocular surface of the two groups was the same, in the order of Proteobacteria, Firmicutes, Actinobacteria, and Bacteroidetes (**Figures 1D,E**). The top six dominant genera in the TAO group (average relative abundance >3.00%) were *Corynebacterium*, *Cutibacterium*, *Staphylococcus*, *Pseudomonas*, *Escherichia-Shigella*, and *Streptococcus*. Among them, *Corynebacterium*, *Cutibacterium*, and *Staphylococcus* were present in all samples of both groups (**Table 2**).

**Table 2:** Features of dominant bacteria.

	Average relative abundance (%)		prevalence (%)		P
The phylum level	TAO	Control	TAO	Control	
Proteobacteria^*^	35.67	18.54	100	100	0.0009
Firmicutes^*^	29.98	39.71	100	100	0.0040
Actinobacteriota^*^	21.99	30.11	100	100	0.0463
*Bacteroidota*	5.84	9.50	100	100	0.6583
*Fusobacteriota*	1.02	0.85	88.06	95.45	0.1085
*Campylobacterota*	1.00	0.10	68.66	77.27	0.1933
Acidobacteriota^*^	0.80	0.09	74.63	36.36	0.0001
Desulfobacterota^*^	0.68	0.18	89.55	81.82	0.0158
Verrucomicrobiota^*^	0.60	0.10	91.04	59.09	0.0025
**the genus level**	**TAO**	**Control**	**TAO**	**Control**	
Corynebacterium^*^	10.13	16.90	100	100	0.0163
*Cutibacterium*	7.15	4.33	100	100	0.7178
*Staphylococcus*	6.68	10.50	100	100	0.0506
*Pseudomonas*	4.79	1.20	98.51	90.91	0.0657
*Escherichia-Shigella*	4.49	2.75	79.10	86.36	0.9429
*Streptococcus*	3.13	1.69	98.51	100	0.7500
*Bacteroides*	2.08	5.10	86.57	81.82	0.7860
*Ligilactobacillus*	1.90	2.49	85.07	77.27	0.2626
*Haemophilus*	1.89	0.23	92.54	100	0.9506
Allorhizobium-Neorhizobium-Pararhizobium-Rhizobium^*^	1.74	0.02	77.61	31.82	0.0000
Bacillus^*^	1.73	0.30	94.03	86.36	0.0043
*Lactobacillus*	1.69	1.49	86.57	77.27	0.4288
*Psychrobacter*	1.58	2.47	98.51	95.45	0.8678
*Salmonella*	1.55	0.54	83.58	90.91	0.6749
*Acetobacter*	1.27	0.60	20.90	22.73	0.9629
Brevundimonas^*^	1.09	0.31	95.52	90.91	0.0048
Acinetobacter^*^	0.95	2.76	98.51	95.45	0.0094
Helicobacter^*^	0.90	0.03	47.76	9.09	0.0011
*Fusobacterium*	0.85	0.55	80.60	95.45	0.0522
*Limosilactobacillus*	0.79	1.22	74.63	68.18	0.3411
Mesorhizobium^*^	0.76	0.01	43.28	13.64	0.0086
*Paracoccus*	0.75	0.64	94.03	90.91	0.9924
*Faecalibacterium*	0.72	3.80	83.58	72.73	0.3254
Micrococcus^*^	0.71	0.89	98.51	100	0.0030
Bifidobacterium^*^	0.70	4.41	88.06	90.91	0.0063
*Pantoea*	0.68	0.23	80.60	77.27	0.1972

TAO, thyroid-associated ophthalmopathy; Control, healthy volunteers; *P < 0.05, Wilcoxon rank sum test.

**Figure 2:**
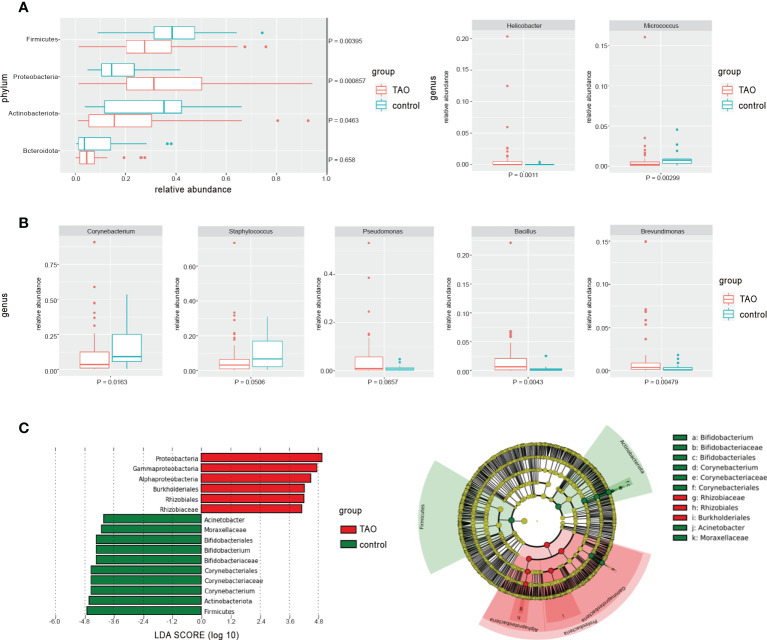
The differences in average relative abundance of dominant bacteria at **(A)** the phylum level and **(B)** the genus level (mean relative abundance > 0.50%) were assessed by the Wilcoxon rank-sum test. **(C)** Linear discriminant analysis was used to verify with significant differences between groups (cutoff = 4.0).

Compared with the control group, the eye surface Proteobacteria abundance in the TAO group was significantly higher. The abundance of Firmicutes and Actinobacteria was lower than that in the control group (P<0.05) (**Figure 2A**). In addition to the common bacterial phyla, Acidobacteriota and Verrucomicrobiota were more prevalent in the ocular surface of patients with TAO, with a significant increase in relative abundance (P<0.05). Among the top 35 bacterial genera with the highest average relative abundance in the TAO group, *Allorhizobium-Neorhizobium-Pararhizobium-Rhizobium*, *Bacillus*, *Brevundimonas*, *Helicobacter*, and *Mesorhizobium* had a significantly increased relative abundance compared with the control group. In contrast*, Corynebacterium*, *Acinetobacter*, *Micrococcus*, and *Bifidobacterium* had a significantly decreased relative abundance (P<0.05) (**Figure 2B** and **Table 2**). The differences in bacterial abundance between the TAO and control groups were also observed in the linear discriminant analysis (cutoff = 4.0) (**Figure 2C**).

### Correlation analysis of clinical manifestations and microbiota

**Figure 3:**
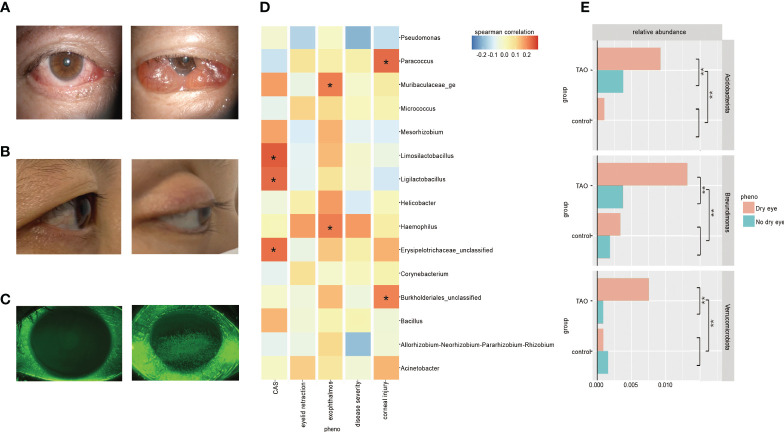
**(A)** Ocular surface symptoms of different disease activity in the thyroid-associated ophthalmopathy (TAO) patients. **(B)** A healthy people (left) and a TAO patient with exophthalmos (right). **(C)** Corneal fluorescein staining results of a healthy person (left) and a TAO patient with moderate corneal epithelial injury (right). **(D)** Bacteria with higher Spearman’s correlation coefficients with CAS, eyelid retraction, exophthalmos, disease severity, and corneal injury in TAO patients are shown at the genus level. **(E)** There were significant differences in the average relative abundance of some bacteria within TAO group and between groups according to dry eyes (t-test dichotomous traits and t-test regression coefficients, P<0.05).

Ocular surface of TAO patients may have orbital pain, eyelid swelling, eyelid erythema, conjunctival redness, chemosis, and inflammation of the caruncle or plica (**Figure 3A** and **Supplementary Table S1**). The score for these symptoms assesses the level of activity in a patient’s disease, with a higher score indicating greater disease activity. This scoring method is called the CAS (Barrio-Barrio et al., 2015). Linear regression analysis showed that Acidobacteriota and Verrucomicrobiota increased with an increase in CAS score; average relative abundance of *Ligilactobacillus*, *Lactobacillus*, *Limosilactobacillus*, and *Bifidobacterium* were positively correlated with CAS (Spearman correlation, <-0.2 or >0.2) (**Figure 3D**).

Exophthalmos, one of the most common clinical manifestations of TAO, is caused by swelling of orbital fat and/or muscle, increasing the area and duration of exposure of the ocular surface to air (**Figure 3B**) (Barrio-Barrio et al., 2015). Linear regression analysis showed that the average relative abundance of *Haemophilus* was positively correlated with exophthalmos (Spearman correlation, <-0.2 or >0.2) (**Figure 3D**).

Both autoimmune inflammatory response and corneal exposure of TAO may cause corneal injury, and the extent of the injury is usually observed by corneal fluorescein staining (**Figure 3C**) (Barrio-Barrio et al., 2015). Linear regression analysis showed that the average relative abundance of *Paracoccus* was positively correlated with corneal epithelial injury (Spearman correlation, <-0.2 or >0.2) (**Figure 3D**).

### Differences in TAO patients with or without dry eye

Clinical changes in soft tissue involvement, exophthalmos, and corneal exposure of TAO patients can increase the risk of dry eye. Previous studies have shown that 65−85% of TAO patients will develop dry eye symptoms (Selter et al., 2015). In this study, the detection rate of dry eye in TAO patients was 76%. The tear breakup time in TAO patients with dry eye (4.35 ± 1.84 s) is significantly lower than that in TAO patients without dry eye (9.02 ± 3.17 s), suggesting an unstable tear film. There was no significant difference in ocular microbiota diversity between TAO dry eye patients and non-dry eye patients (P>0.05). The abundance of Acidobacteriota, Verrucomicrobiota, *Brevundimonas*, and *Acinetobacter* in dry eye patients in the TAO group was significantly higher than that in non-dry eye patients. Among them, Acidobacteriota, Verrucomicrobiota, and *Brevundimonas* increased significantly in the ocular surface abundance of TAO patients with dry eye, which was significantly higher than that of TAO patients without dry eye, healthy individuals (control) with dry eye, and healthy individuals (control) without dry eye (P<0.05) (**Figure 3E**).

### Traditional culture

Among the 43 patients with TAO, 29 samples were successfully cultured with one or more bacteria, and 11 different species of bacteria were detected. Bacteria were cultured in 17 of 43 healthy subjects under the same culture conditions, and a total of six bacteria were detected (**Supplementary Table S2**). *Staphylococcus epidermidis* accounted for more than half of the culture results, which were 69.0% and 64.7% in the TAO and control groups, respectively. The detection rate of *Cutibacterium acnes* in the TAO group (20.7%) was significantly higher than that in the control group (5.9%). TAO group belonging to *Corynebacterium* are *Corynebacterium accolens*, *Corynebacterium macginleyi*, *Corynebacterium suicordis*, and *Corynebacterium tuberculostearicum*; belonging to *Staphylococcus* are *Staphylococcus epidermidis*, *Staphylococcus haemolyticus*, and *Staphylococcus aureus*. In addition, *Bacillus cereus*, *Enterococcus faecalis*, and *Stenotrophomonas maltophilia* were also detected.

## Discussions

The study of ocular surface microbiota by high-throughput sequencing technology can reveal the diversity of ocular surface microbiota of patients more comprehensively; discover the types, average relative abundance, and changes in bacteria involved in the composition of ocular surface flora. By searching previous high-throughput sequencing studies of ocular surface microbiota, it was found that the dominant bacterial phyla were Proteobacteria, Actinobacteria, Firmicutes, and Bacteroides (Dong et al., 2011; Huang et al., 2016; Doan et al., 2016; Ozkan et al., 2018; Li, Gong, et al., 2019; Dong et al., 2019; Li, Yi, et al., 2019; Yau et al., 2019; Ozkan et al., 2019; Kang et al., 2020; Yan et al., 2020; Shivaji et al., 2021) (**Supplementary Table S3**), while the dominant genera were *Corynebacterium*, *Pseudomonas*, *Staphylococcus*, *Acinetobacter*, *Streptococcus*, and *Cutibacterium* (Dong et al., 2011; Huang et al., 2016; Doan et al., 2016; Ozkan et al., 2018; Li, Gong, et al., 2019; Dong et al., 2019; Li, Yi, et al., 2019; Yau et al., 2019; Ozkan et al., 2019; Kang et al., 2020; Yan et al., 2020; Shivaji et al., 2021) (**Supplementary Table S4**). In addition, *Bacillus* (Li, Gong, et al., 2019) (Li, Yi, et al., 2019; Yan et al., 2020) and *Escherichia* (Ozkan et al., 2019; Shivaji et al., 2021) have been noted to be the dominant bacteria in some studies. The results of this study showed that the dominant bacterial phyla in the TAO group were Proteobacteria, Firmicutes, Actinobacteria, and Bacteroidetes. The top six dominant bacterial genera were *Corynebacterium*, *Cutibacterium*, *Staphylococcus*, *Pseudomonas*, *Escherichia-Shigella*, and *Streptococcus*, which was consistent with the results of previous studies on ocular surface dominant microbiota. However, compared with healthy individuals, the average relative abundance of many bacteria on the ocular surface of TAO patients, including these dominant bacteria, was significantly changed.

As the dominant bacteria on the ocular surface, *Corynebacterium* had the highest detection rate and average relative abundance in both groups in this study. However, compared with the control group, the average relative abundance of *Corynebacterium* in the TAO group was significantly decreased (P<0.05), which also occurred in traumatic corneal ulcer (Kang et al., 2020) and bacterial keratitis (Shivaji et al., 2021) patients with significant ocular surface changes. Some studies found that there was a negative correlation between *Corynebacterium* and bacteria of other genera (St Leger et al., 2017). *Corynebacterium* participates in the formation of normal ocular surface microbiome homeostasis. Ocular surface diseases may destroy ocular surface structure or significantly increase pathogenic bacteria and opportunistic pathogens, which may lead to decreased average relative abundance of *Corynebacterium*.

TAO patients often experience photophobia, eye irritation, itching, and foreign body sensation in the eyes. The typical clinical manifestations of TAO patients include hyperemia and edema of the conjunctiva and lacrimal plica. These symptoms are very similar to conjunctivitis, but previous studies thought they were caused by a single immune factor. Notably, the sequencing and culture results of the present study suggest that opportunistic pathogens may be involved in this process. The results of sequencing showed that the average relative abundance of *Bacillus* and *Brevundimonas* increased significantly, especially the increase of *Brevundimonas* in the ocular surface of TAO patients with dry eye disease, *Paracoccus* was positively correlated with corneal epithelial injury, and *Haemophilus* was positively correlated with exophthalmia in TAO patients (P<0.05). Among them, *Bacillus* is one of the main causes of traumatic endophthalmitis (Teweldemedhin et al., 2017). *Brevundimonas diminuta* can cause secondary keratitis (Burch et al., 2021). Previous studies have shown that the average relative abundance of *Paracoccus* was significantly increased in patients with bacterial keratitis (Shivaji et al., 2021), and *Paracoccus yeei* can appear as pathogenic bacteria in keratitis induced by wearing contact lenses (Courjaret et al., 2014), infection complicated after penetrating keratoplasty (Kanis et al., 2010), and uveitis of unknown etiology (negative culture) (Drancourt et al., 2008). *Haemophilus* can be detected in conjunctivitis, keratitis, blepharitis, dacryocystitis, and endophthalmitis (Alrawi et al., 2002; Tabuenca Del Barrio et al., 2021). The results of bacterial culture further confirmed the existence of ocular surface pathogens in TAO patients. Culture results showed that a large number of opportunistic pathogens could be detected on the ocular surface of TAO patients. Among them, *Staphylococcus aureus* is a common cause of bacterial keratitis, and its corneal toxicity has been proven (Putra et al., 2019; Caballero et al., 2021). *Stenotrophomonas maltophilia* and *Corynebacterium macginleyi* were detected in multiple cases of bacterial keratitis (Cho and Lee, 2021; Sagerfors et al., 2021). *Bacillus cereus*, *Enterococcus faecalis*, *Staphylococcus haemolyticus* and *cutibacterium acnes* are considered as opportunistic pathogens of endophthalmitis (Panda and Singh, 2018; Ehling-Schulz et al., 2019; Chen et al., 2021; Fowler et al., 2021). In the culture results of the control group, only two opportunistic pathogens were found, among which *Acinetobacter lwoffii* could induce endophthalmitis after cataract surgery (Roy et al., 2015). The other, *cutibacterium acnes*, can cause postoperative endophthalmitis (Fowler et al., 2021) and keratitis (Ovodenko et al., 2009), but its detection rate on the ocular surface of TAO patients is significantly higher. In previous studies of TAO-exposed keratitis, successful bacterial culture include *Pseudomonas* sp., *Acinetobacter* sp., *Corynebacterium* sp., *Escherichia coli*, *Streptoccocus pneumoniae*, *Staphylococcus aureus* and *Staphylococcus epidermidis*, some of which were consistent with the results of sequencing and culture in this study (Naik et al., 2019). This suggests that the presence or increased average relative abundance of these potential pathogens increases the potential risk of ocular surface infection in non-exposed keratitis TAO patients.

Due to the limited culture conditions provided by the laboratory, the positive rate of traditional culture was lower, while the 16S rDNA amplicon sequencing technique can detect more kinds of bacteria more sensitively. In this study, the positive rate of sequencing reached 100%, and 756 bacteria were found at the “genus” level, but the positive rate of culture was 67.4%, and 11 bacterial strains were found. Even in previous studies of TAO-exposed keratitis, 14.3% of samples were not successfully cultured (Naik et al., 2019). The findings of the present study suggest that part of the sequencing results was consistent with the culture. The dominant bacteria, *Corynebacterium* (average relative abundance, 10.13%) and *Staphylococcus* (average relative abundance, 6.68%), in all ocular surface samples of TAO, are easier to obtain pure strains under suitable conditions, and the detection rates of *Corynebacterium* and *Staphylococcus* were 36.4% and 27.3%, respectively. These two species have also been detected in previous studies on TAO-exposed keratitis, with the detection rates of *Corynebacterium* and *Staphylococcus* being 25% and 17.7%, respectively (Naik et al., 2019). However, only Brain-Heart Infusion medium was used in this study, which could not meet the growth conditions of other TAO ocular surface dominant bacteria, such as *Cutibacterium*, *Pseudomonas*, and *Escherichia-Shigella*. The culture results reflect the reliability of the 16S rDNA amplicon sequencing technique and also suggest that a more suitable environment for the growth of ocular surface bacteria can be designed, according to the sequencing results, to improve the culture-positive rate.

The study of the natural course of TAO suggested that the onset of TAO can be followed by a progressive and worsening autoimmune response lasting several months, with pathological manifestations of orbital tissue inflammation, lymphocyte infiltration, glycoamine production, and edema (Bartalena et al., 2020). This phase is referred to as active TAO, and whether TAO patients are active can be determined by evaluating CAS (Bartalena et al., 2020). This study found that the average relative abundance of *Ligilactobacillus*, *Lactobacillus*, *Limosilactobacillus*, and *Bifidobacterium* in TAO patients was positively correlated with CAS (P<0.05). Previous studies have suggested that some *Lactobacillus* and *Bifidobacterium* species have significant structural homology with thyroid peroxidase and anti-thyroglobulin and can competitively bind thyroid peroxidase antibodies and anti-thyroglobulin antibodies to induce autoimmune thyroid disease through molecular mimicry (Kiseleva et al., 2011; Benvenga and Guarneri, 2016). Notably, the thyroid peroxidase antibody level of TAO patients is significantly correlated with the disease and plays an important role in autoimmune disease (Lantz et al., 2014; Lee et al., 2014; Godlewska et al., 2019).

The ocular microflora of TAO patients may be directly involved in the disease through cross-reaction induced by autoantigens and may also affect the ocular microenvironment through metabolites. The surface of the eye is covered with tear film, with an approximate pH of 7.45, ranging from 7.14 to 7.82 (Van Haeringen, 1981). ASIC1, ASIC3, and isomer ASIC1/ASIC3 channels in the distal eye meridian are activated when the pH of the ocular surface is between 6.5 and 7.2, resulting in ocular discomfort (Comes et al., 2021). The bacteria with high abundance in this study, which were positively correlated with CAS, can produce a variety of acid metabolites (Mahlen and Clarridge, 2009). These acid metabolites are likely to change the composition of eye surface tear, reduce the PH of tear, and cause early clinical manifestations, such as non-specific eye irritation, itching, and foreign body sensation in TAO patients. However, whether ocular surface microbiota is involved in the occurrence and development of diseases through these pathways still needs further research to verify.

This study found no significant difference in diversity between the TAO and control groups, which was similar to allergic rhinoconjunctivitis (Yau et al., 2019), Sjögren’s syndrome (de Paiva et al., 2016), and conjunctival mucosa-associated lymphoid tissue lymphoma (Asao et al., 2019). The reason for this result may be related to the small sample size of the control group in this study, which reduced the statistical power. Another possibility is that the participants in the control group were older than those in the TAO group. Previous studies have found that the conjunctival microbiota diversity is higher in healthy older adults (47−84 years of age) than in young people (23−44 years of age) (Wen et al., 2017). Although most of the subjects in the present study were elderly, age difference may still be one of the reasons for the insignificant difference between the two groups.

In conclusion, this study found that the composition of ocular microbiota in patients with TAO was significantly different from that in healthy individuals. The presence of opportunistic pathogen increases the risk of ocular surface infection. The findings of this study provide a new avenue of research into the mechanism of ocular surface in TAO patients.

## Data availability statement

The datasets presented in this study can be found in online repositories. The names of the repository/repositories and accession number(s) can be found in the article/**supplementary material**.

## Ethics statement

Written informed consent was obtained from the individual(s) for the publication of any potentially identifiable images or data included in this article.

## Author contributions

BS and JX supervised this study. XJ, KD and ZZ conceived and designed the experiments. XJ, XN, QM and ZK collected the samples. JP, JY and XJ analyzed the data. XJ, KD and BS wrote the manuscript. The final manuscript was read and approved by all authors.

## Funding

This work was supported by grants from the Research Units of Discovery of Unknown Bacteria and Function (2018RU010); the State Key Laboratory of Infectious Disease Prevention and Control (2019SKLID313) and (2019SKLID312); the Scientific Research Project of Shanxi Provincial Health Commission (2019076), (2020XM07), (2020SYS12) and (2021XM46); the Natural Science Research Project of Shanxi Provincial Department of Science and Technology (201903D311009), (20210302123343) and (202104010910013); the Shanxi Provincial Eye Hospital Innovation Fund Project (C201901).

## Acknowledgments

We express our sincere thanks to Shanxi Eye Hospital, the State Key Laboratory of Infectious Disease Prevention and Control and all of the participants who recruited patients in this study.

## Conflict of interest

The authors declare that the research was conducted in the absence of any commercial or financial relationships that could be construed as a potential conflict of interest.

## Publisher’s note

All claims expressed in this article are solely those of the authors and do not necessarily represent those of their affiliated organizations, or those of the publisher, the editors and the reviewers. Any product that may be evaluated in this article, or claim that may be made by its manufacturer, is not guaranteed or endorsed by the publisher.
