# Supplementary material for: Comparison of the ocular surface microbiota between thyroid-associated ophthalmopathy patients and healthy subjects
Source: Front Cell Infect Microbiol. 2022 Jul 26;12:914749. doi: 10.3389/fcimb.2022.914749 (PMC9360483; doi:10.3389/fcimb.2022.914749)
Supplement: Supplementary file 1 [file Table_1.doc]

**Supplementary Table S1 Clinical data of TAO patients**

| **Sample number** | **age** | **sex** | **dry eye/**  **no dry eye** | **Clinical Activity Score** | **eyelid retraction**  **(mm)** | **Exophthalmos**  **(mm)** | **Severity Degree** | **tear breakup time(s)** | **corneal fluorescein staining** |
| --- | --- | --- | --- | --- | --- | --- | --- | --- | --- |
| T02 | 45 | Male | dry eye | 2 | 0 | 14 | mild | 2.48 | normal |
| T03 | 47 | Female | dry eye | 3 | 3 | 20 | moderate-severe | 3.31 | normal |
| T04 | 48 | Male | dry eye | 3 | 1 | 20 | mild | 6.37 | trace |
| T06 | 39 | Male | dry eye | 3 | 4 | 17 | moderate-severe | 2.48 | mild |
| T12 | 57 | Male | no dry eye | 3 | 0 | 15 | mild | 9.24 | normal |
| T14 | 48 | Female | no dry eye | 3 | 0 | 20 | mild | 5.93 | normal |
| T15 | 51 | Female | dry eye | 4 | 1 | 20 | mild | 8.22 | trace |
| T16 | 46 | Male | no dry eye | 2 | 0 | 18 | mild | 6.56 | normal |
| T19 | 53 | Male | no dry eye | 3 | 1 | 17 | mild | 6.31 | normal |
| T20 | 31 | Female | no dry eye | 3 | 3 | 13 | mild | 8.99 | normal |
| T22 | 52 | Female | dry eye | 3 | 1 | 14 | mild | 3.06 | normal |
| T23 | 52 | Female | dry eye | 4 | 3 | 23 | moderate-severe | 5.35 | mild |
| T24 | 59 | Male | dry eye | 3 | 1 | 20 | mild | 4.4 | mild |
| T26 | 34 | Female | dry eye | 3 | 0 | 16 | mild | 4.21 | normal |
| T28 | 52 | Male | dry eye | 3 | 0 | 17 | mild | 4.76 | normal |
| T29 | 33 | Male | dry eye | 4 | 0 | 17 | mild | 3.7 | trace |
| T30 | 52 | Female | dry eye | 3 | 1 | 20 | mild | 4.08 | normal |
| T31 | 28 | Male | dry eye | 3 | 1 | 19 | mild | 3.25 | trace |
| T32 | 52 | Female | dry eye | 3 | 1 | 16 | mild | 5.42 | trace |
| T33 | 63 | Male | dry eye | 3 | 2 | 21 | moderate-severe | 2.87 | normal |
| T34 | 48 | Female | dry eye | 2 | 1 | 13 | mild | 3.12 | mild |
| T35 | 48 | Male | no dry eye | 3 | 2 | 22 | moderate-severe | 9.56 | normal |
| T36 | 27 | Female | dry eye | 3 | 0 | 15 | mild | 3.25 | normal |
| T37 | 72 | Male | dry eye | 3 | 0 | 15 | mild | 4.01 | severe |
| T38 | 50 | Female | dry eye | 4 | 5 | 18 | moderate-severe | 6.56 | mild |
| T39 | 21 | Female | dry eye | 4 | 4 | 17 | moderate-severe | 3.44 | normal |
| T41 | 28 | Male | dry eye | 2 | 1 | 18 | mild | 3.31 | normal |
| T42 | 45 | Female | dry eye | 3 | 2 | 22 | moderate-severe | 7.65 | moderate |
| T43 | 49 | Male | dry eye | 2 | 2 | 14 | mild | 7.46 | mild |
| T44 | 61 | Female | dry eye | 3 | 1 | 12 | mild | 3.82 | normal |
| T45 | 56 | Male | dry eye | 4 | 2 | 25 | moderate-severe | 6.5 | mild |
| T46 | 32 | Female | no dry eye | 2 | 0 | 17 | mild | 6.88 | normal |
| T47 | 14 | Female | no dry eye | 2 | 2 | 18 | moderate-severe | 14.34 | trace |
| T48 | 50 | Female | dry eye | 4 | 3 | 23 | moderate-severe | 5.35 | moderate |
| T50 | 67 | Male | dry eye | 2 | 0 | 16 | mild | 4.46 | moderate |
| T51 | 68 | Female | dry eye | 3 | 2 | 15 | mild | 1.72 | mild |
| T52 | 40 | Female | dry eye | 2 | 1 | 16 | mild | 2.68 | trace |
| T53 | 47 | Female | dry eye | 3 | 1 | 18 | mild | 2.29 | trace |
| T57 | 39 | Female | no dry eye | 2 | 1 | 15 | mild | 5.8 | normal |
| T59 | 40 | Female | dry eye | 2 | 1 | 18 | mild | 4.46 | mild |
| T61 | 23 | Female | dry eye | 2 | 3 | 22 | moderate-severe | 4.4 | normal |
| T62 | 16 | Male | no dry eye | 3 | 0 | 16 | mild | 9.75 | normal |
| T63 | 42 | Female | dry eye | 3 | 0 | 20 | mild | 3.06 | mild |
| T64 | 20 | Female | dry eye | 4 | 3 | 20 | moderate-severe | 9.56 | mild |
| T65 | 48 | Male | no dry eye | 3 | 2 | 21 | moderate-severe | 5.16 | normal |
| T66 | 43 | Male | dry eye | 4 | 2 | 18 | moderate-severe | 6.69 | mild |
| T70 | 56 | Female | dry eye | 3 | 1 | 15 | mild | 3.63 | mild |
| T71 | 21 | Female | dry eye | 2 | 2 | 16 | mild | 3.82 | mild |
| T72 | 61 | Male | no dry eye | 4 | 0 | 14 | mild | 8.73 | normal |
| T73 | 34 | Male | dry eye | 3 | 0 | 16 | mild | 4.21 | normal |
| T76 | 42 | Male | no dry eye | 2 | 0 | 14 | mild | 14.21 | normal |
| T77 | 46 | Female | no dry eye | 4 | 2 | 17 | moderate-severe | 12.81 | normal |
| T78 | 52 | Male | dry eye | 4 | 1 | 14 | mild | 3.44 | normal |
| T79 | 22 | Female | dry eye | 2 | 0 | 16 | mild | 2.29 | normal |
| T80 | 53 | Female | dry eye | 4 | 3 | 17 | moderate-severe | 6.37 | moderate |
| T81 | 50 | Male | dry eye | 5 | 4 | 14 | moderate-severe | 4.4 | severe |
| T82 | 39 | Female | dry eye | 3 | 2 | 20 | moderate-severe | 2.48 | mild |
| T83 | 54 | Female | dry eye | 3 | 0 | 12 | mild | 2.68 | normal |
| T84 | 24 | Female | no dry eye | 3 | 0 | 16 | mild | 13.57 | mild |
| T85 | 39 | Female | dry eye | 2 | 0 | 17 | mild | 5.42 | mild |
| T86 | 47 | Male | dry eye | 5 | 1 | 17 | moderate-severe | 9.18 | mild |
| T87 | 41 | Male | dry eye | 4 | 4 | 24 | moderate-severe | 3.25 | mild |
| T88 | 29 | Female | dry eye | 2 | 1 | 14 | mild | 4.59 | normal |
| T89 | 58 | Female | no dry eye | 5 | 0 | 16 | mild | 6.5 | normal |
| T90 | 49 | Female | dry eye | 3 | 0 | 17 | mild | 3.12 | mild |
| T91 | 72 | Female | dry eye | 4 | 0 | 19 | mild | 2.55 | normal |
| T92 | 40 | Male | dry eye | 3 | 3 | 21 | moderate-severe | 2.74 | moderate |
