# Supplementary material for: Comparison of the ocular surface microbiota between thyroid-associated ophthalmopathy patients and healthy subjects
Source: Front Cell Infect Microbiol. 2022 Jul 26;12:914749. doi: 10.3389/fcimb.2022.914749 (PMC9360483; doi:10.3389/fcimb.2022.914749)
Supplement: Supplementary file 2 [file Table_2.doc]

**Supplementary Table S2 Statistical tables of bacteria isolated and cultured on the ocular surface**

| **Group** | **Serial number** | **Species** | **Genus** | **Number of isolated bacteria** |
| --- | --- | --- | --- | --- |
| TAO | 1 | *Bacillus cereus* | *Bacillus* | 2 |
|  | 2 | *Corynebacterium accolens* | *Corynebacterium* | 1 |
|  | 3 | *Corynebacterium macginleyi* | *Corynebacterium* | 4 |
|  | 4 | *Corynebacterium suicordis* | *Corynebacterium* | 1 |
|  | 5 | *Corynebacterium tuberculostearicum* | *Corynebacterium* | 1 |
|  | 6 | *Cutibacterium acnes* | *Cutibacterium* | 6 |
|  | 7 | *Enterococcus faecalis* | *Enterococcus* | 1 |
|  | 8 | *Staphylococcus epidermidis* | *Staphylococcus* | 20 |
|  | 9 | *Staphylococcus haemolyticus* | *Staphylococcus* | 1 |
|  | 10 | *Staphylococcus aureus* | *Staphylococcus* | 1 |
|  | 11 | *Stenotrophomonas maltophilia* | *Stenotrophomonas* | 1 |
| Control | 1 | *Acinetobacter lwoffii* | *Acinetobacter* | 1 |
|  | 2 | *Corynebacterium accolens* | *Corynebacterium* | 1 |
|  | 3 | *Cutibacterium acnes* | *Cutibacterium* | 1 |
|  | 4 | *Saccharopolyspora endophytica* | *Saccharopolyspora* | 1 |
|  | 5 | *Staphylococcus epidermidis* | *Saccharopolyspora* | 11 |
|  | 6 | *Staphylococcus warneri* | *Staphylococcus* | 2 |
