# Supplementary material for: Comparison of the ocular surface microbiota between thyroid-associated ophthalmopathy patients and healthy subjects
Source: Front Cell Infect Microbiol. 2022 Jul 26;12:914749. doi: 10.3389/fcimb.2022.914749 (PMC9360483; doi:10.3389/fcimb.2022.914749)
Supplement: Supplementary file 3 [file Table_3.doc]

| **Supplementary Table S3 Average relative abundance of dominant bacteria at phylum level（%）** | | | | | | |
| --- | --- | --- | --- | --- | --- | --- |
| **References** | **Object** | Group | ***Proteobacteria*** | ***Actinobacteria*** | ***Firmicutes*** | ***Bacteroidetes*** |
| (Huang et al., 2016) | normal | — | 46.5 | 33.89 | 15.5 | 2.28 |
| (Dong et al., 2011) | healthy | — | 64 | 19.6 | 3.9 | 0.16 |
| (Ozkan et al., 2019) | pterygium^*^ | — | 44.6 | 8.7 | 36.3 | ND |
| (Kang et al., 2020) | Traumatic corneal ulcer | case | 75.61 | 2.37 | 13.0 | 3.05 |
|  |  | control | 28.35 | 29.61 | 35.72 | 1.35 |
| (Yan et al., 2020) | blepharitis ciliaris | case | 20.51 | 20.66 | 41.71 | 7.95 |
|  |  | control | 26.96 | 23.87 | 29.30 | 11.04 |
| (Li et al., 2019b) | xerophthalmia | case | 47.62 | 6.24 | 17.2 | 16.54 |
|  |  | control | 51.7 | 6.12 | 16.86 | 13.6 |
| (Dong et al., 2019) | Meibomian gland dysfunction | case | 27.46 | 34.17 | 31.70 | 2.21 |
|  |  | control | 14.66 | 56.98 | 19.67 | 3.30 |
| (Li et al., 2019a) | diabetes | case | 48.86 | 6.43 | 18.04 | 15.83 |
|  |  | control | 56.76 | 6.16 | 15.66 | 10.07 |
| (Shivaji et al., 2021) | bacterial coronitis | case | 42.78 | 15.76 | 39.56 | 0.31 |
|  |  | control | 11.37 | 25.45 | 28.75 | 0.29 |
| (Yau et al., 2019) | Allergic conjunctivitis | † | 41.07 | 15.81 | 30.55 | 8.12 |

Table S1. "ND", no data; *, Swabs were used to obtain non-operative site conjunctiva surface samples for patients undergoing pterygium surgery; †: The mean value of case group and control group.
