# Supplementary material for: Comparison of the ocular surface microbiota between thyroid-associated ophthalmopathy patients and healthy subjects
Source: Front Cell Infect Microbiol. 2022 Jul 26;12:914749. doi: 10.3389/fcimb.2022.914749 (PMC9360483; doi:10.3389/fcimb.2022.914749)
Supplement: Supplementary file 4 [file Table_4.doc]

| **Supplementary Table S4** **Average relative abundance of dominant bacteria at genus level (%)** | | | | | | | | |
| --- | --- | --- | --- | --- | --- | --- | --- | --- |
| **References** | **Object** | Group | ***Corynebacterium*** | ***Pseudomonas*** | ***Staphylococcus*** | ***Acinetobacter*** | ***Streptococcus*** | ***Cutibacterium*** |
| (Dong et al., 2011) | healthy | — | 15 | 20 | 4 | 12 | 1 | 20 |
| (Doan et al., 2016) | healthy | — | 14.2 | 27.83 | 13.2 | 0.55 | 4.39 | 8.24 |
| (Ozkan et al., 2019) | pterygium* | — | 4 | 2.1 | 3 | 2.2%–3.8%† | 2.3 | ND |
| (Huang et al., 2016) | normal | — | 28.22 | 26.75 | 5.28 | 4.74 | 2.85 | ND |
| (Ozkan et al., 2018) | pterygium* | — | 13.50 | 6.30 | ND | 4.3 | 4.80 | ND |
| (Kang et al., 2020) | Traumatic corneal ulcer | case | 0.53 | 57.49 | 2.25 | ND | 4.24 | ND |
|  |  | control | 19.42 | 0.47 | 3.83 | ND | 22.93 | ND |
| (Yan et al., 2020) | blepharitis ciliaris | case | 5.93 | 1.99 | ND | 2.69 | 1.13 | 1.26 |
|  |  | control | 5.47 | 1.88 | ND | 3.9 | 0.85 | 2.83 |
| (Li et al., 2019b) | xerophthalmia | case | 2.61 | 11.49 | ND | 7.79 | ND | ND |
|  |  | control | 2.73 | 17.73 | ND | 8.81 | ND | ND |
| (Dong et al., 2019) | Meibomian gland dysfunction | case | 20.22 | ND | 20.71 | ND | 2.8 | 9.29 |
|  |  | control | 46.43 | ND | 7.88 | ND | 3.89 | 5.44 |
| (Li et al., 2019a) | diabetes | case | 2.94 | 13.91 | 2.4 | 7.30 | 0.8 | ND |
|  |  | control | 2.18 | 23.33 | 1.0 | 11.09 | 0.9 | ND |
| (Shivaji et al., 2021) | bacterial coronitis | case | 10.95 | 0.56 | 16.05 | 0.25 | 27.7 | 0.17 |
|  |  | control | 3.91 | 5.71 | 2.89 | 1.15 | 7.33 | 0.05 |
| (Yau et al., 2019) | Allergic conjunctivitis | ‡ | 4.57 | ND | 10.54 | ND | 13.86 | ND |

Table S2. "ND", no data; *: Swabs were used to obtain non-operative site conjunctiva surface samples for patients undergoing pterygium surgery; †: The genus zOTUs assigned to the genus Acinetobacter (zOTU79, zOTU239); ‡: The mean value of case group and control group.
